# Arrhenotoky and oedipal mating in the northern fowl mite (*Ornithonyssus sylviarum*) (Acari: Gamasida: Macronyssidae)

**DOI:** 10.1186/1756-3305-5-281

**Published:** 2012-12-04

**Authors:** John B McCulloch, Jeb P Owen

**Affiliations:** 1 Department of Entomology, Washington State University, PO Box 646382, Pullman, WA 99164, USA; 2 Center for Reproductive Biology, Washington State University, Pullman, WA 99164, USA

**Keywords:** Sex-Ratio, Reproduction, Ectoparasite, Transmission, Poultry

## Abstract

**Background:**

The northern fowl mite (NFM; *Ornithonyssus sylviarum*) is a blood-feeding ectoparasite of birds and a major pest of poultry in the United States. Mite populations spread rapidly in commercial flocks, reach peak burdens of >70,000 mites per bird and have developed resistance to many pesticides. Despite decades as a pest in the United States, the reproductive biology of NFM remains unclear. Based on karyotypes, the NFM has haplodiploid sex determination, which suggests unmated females could produce male offspring (arrhenotoky). Thus, unmated females could disseminate to a new host and initiate an infestation by producing and mating with sons (oedipal mating).

**Methods:**

We used small capsules to isolate and recover NFM on host chickens. Mites in capsules could blood feed, develop and reproduce, but could not contact other mites. Individual larvae were matured in isolation to produce known, unmated females. We evaluated reproduction of (I) previously mated females (i) in isolation, or (ii) paired with a male, and (II) unmated (virgin) females in isolation. In each treatment we recorded the number and sexes of offspring produced over time.

**Results:**

Mated NFM produced female and male offspring in isolation, or when paired with a male. When paired with a male, females produced a female-biased sex ratio of the offspring (F:M ratio ~5:1). Unmated, female NFM produced exclusively male offspring when in isolation. When paired with their sons that had developed to maturity, the "virgin" females were able to mate and subsequently produce female offspring.

**Conclusions:**

This study found that females with immediate access to sperm produced mostly female offspring. Virgin female NFM initially produced only male offspring and subsequently used oedipal mating to produce female offspring. Using this reproductive system NFM could successfully colonize new hosts as immature, or unmated females. The strong female-biased sex ratio of NFM populations suggests a large proportion of the parasite population is capable of disseminating to new hosts, which is essential for an obligate parasite to persist.

## Background

The northern fowl mite (*Ornithonyssus sylviarum,* NFM) (Canestrini and Fanzago, 1877) is a blood-feeding ectoparasite of birds that is a common pest of the poultry industry in North America [1]. Infestations by NFM on laying hens reach high densities (>70,000 mites/bird), which result in host blood loss, tissue inflammation and costly reductions in productivity [1,2]. Mite infestations spread quickly through commercial flocks and are difficult to control due to pesticide resistance [1,3]. The sex ratios of NFM populations are female biased (~80%) [4], suggesting that genetic, or symbiotic (e.g. *Wolbachia*) mechanisms influence sex determination [5,6]. Through karyotyping, Oliver [7] demonstrated that NFM are haplodiploid, which provides a genetic mechanism for sex ratio manipulation. In arrhenotoky males arise from unfertilized eggs and are haploid, whereas females arise from fertilized eggs and are diploid. Alternatively, a pseudo-arrhenotokous system requires egg fertilization and subsequent elimination of the paternal genome produces haploid males [5]. Oliver [7] reported that captured NFM females (presumed to be mated) produced eggs in a 1:1 haploid-diploid ratio and virgin female NFM produced only haploid eggs. Oliver did not determine the mating status of NFM females, nor confirm if haploid eggs developed into male mites. Thus, it has remained unknown if fertilization is required to produce male offspring by NFMs.

An important rationale for determining if NFM is arrhenotokous relates to parasite dispersal. The persistence of parasites is reliant on successful dispersal (transmission) to new hosts [8,9]. For sexually reproducing parasites, mating can constrain dispersal if the parasite must be mated before colonizing a new host or both sexes need to disperse together. Arrhenotoky alleviates constraints on dispersal through oedipal mating (i.e. mating with sons) [10]. Virgin females can produce and mate with male offspring (sons), which enables the production of females. This is particularly advantageous for parasites, because it allows females to colonize new hosts as juveniles or as unmated adults without males [10].

Determining if NFM is arrhenotokous also provides insight into the evolution of arrhenotoky among arthropods. For example Cruickshank and Thomas [11] used a phylogenetic analysis of mites to test the hypothesis that arrhenotoky evolves from pseudo-arrhenotoky. Their phylogeny included Macronyssidae. However, NFM was absent in the dataset, because the sex determination system has remained undetermined. Haplodiploid genetic systems have also been the focus of evolutionary questions surrounding competition for mates, local adaptation and the loss of deleterious alleles [12-14]. Here too NFM is a useful system, because the mites are strongly aggregated on the host [15], have adapted to highly diverse host taxa [16] and effectively adapt to pesticide pressure [3].

We used on-host isolation of female NFM to control mating history and to test if NFM produce male offspring via arrhenotoky. We report the effects of mating on offspring sex ratio and describe the importance of NFM sex-determination to the spread and persistence of this ectoparasite.

## Methods

### Mite colonies

The NFMs were obtained from a colony at the University of California, Riverside [2]. Mites were maintained on female, white leghorn chickens (*Gallus gallus*) (Linnaeus, 1758). Birds were caged individually and were infested at 18 weeks of age, or older. Cages were housed in temperature and light-controlled rooms (24°C; 14:10 L:D). Chickens used in this study were cared for in accordance with the regulations of IACUC (protocol #03736) and the Office of the Campus Veterinarian at Washington State University. Each bird was used as a host for no longer than 2 weeks to avoid possible effects of host immune responses on NFM survival and reproduction [2].

### Capsules and mite isolation

**Figure 1:**
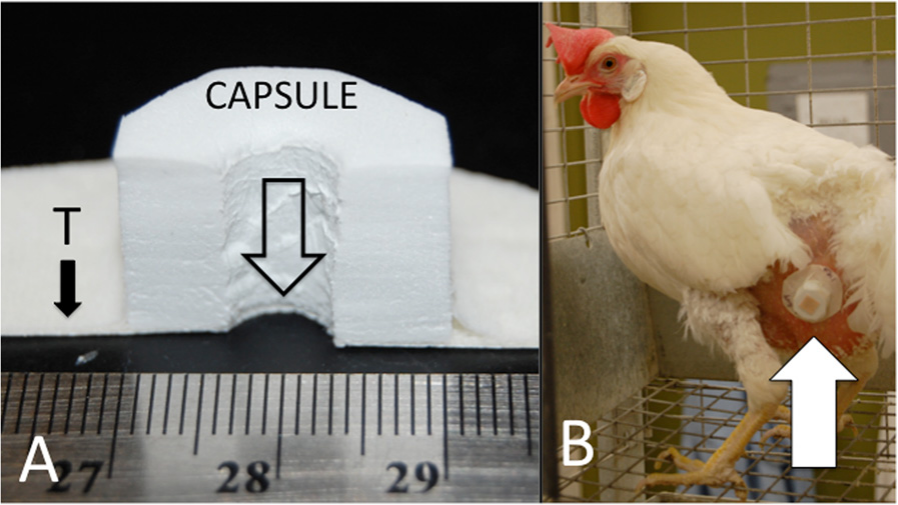
**Design and use of the capsule to isolate mites on-host**. (**A**) A cross-section of a solid foam capsule attached to double-sided tape (T), which was used to attach the capsule to the bird's skin. A hole bored through the foam (open arrow) enclosed the mite(s), but allowed access to the skin for blood feeding. (**B**) An intact capsule attached to a hen (white arrow) on the lower abdomen. Feathers were cleared from the area to attach the capsule.

Capsules were constructed using closed-cell foam cylinders approximately 2.5 cm diameter X 2 cm tall (Figure 1a). Cylinders were attached to one side of a piece of ACE™ double-sided, fabric carpet tape (5 cm × 36 mm). A skirting of paper medical tape (Johnson and Johnson™) was wrapped around the border between the foam cylinder and the carpet tape to prevent chicken dander from weakening the contact between the capsule and the tape. The exposed adhesive on the upper side of the carpet tape was also covered with paper medical tape to prevent feathers from adhering to the surface. A cork borer (outside diameter 8 mm) was used to bore a hole through the foam cylinder and carpet tape base. The hole through the capsule allowed mites access to the skin surface for blood feeding and provided an interior space where mites could reside and oviposit. The adhesive base of the capsule was placed on a patch of skin trimmed free of feathers on the lateral side of the lower abdomen (Figure 1b). Mites were placed into the capsule using a fine brush and the opening of the capsule was sealed with a round adhesive bandage.

Capsules (2 per bird) remained attached to a bird for up to 2 weeks. The mites were collected from the capsule interior using a brush or aspirator. Eggs in the capsule, which were visible under a dissecting microscope, were carefully moved into glass vials (0.5 dram) using a fine brush or were left inside the capsule. Mite eggs (in vials or capsules) were hatched in an incubator (26°C, 96% humidity maintained by saturated KNO_3_ solution). Protonymphs were isolated in capsules on the host for six days to blood feed and molt to the adult stage. Adult mites were recovered from the capsules and sexed according to modified methods of Crystal [17], replacing 50% ethanol solution with 1x phosphate buffered saline. Mites in the experiments were either (i) captured from on-host infestations as adults, or (ii) reared from the egg stage in isolation (on-host capsules). Capsule-reared mites were un-mated, because they remained in isolation through development. Capsule-reared female mites were considered “virgin” in the experiments described below. Adult mites collected from intact on-host populations were assumed to be mated.

### Experiment 1: determining the effect of mating opportunity on sex of NFM offspring

Female mites were placed into on-host capsules in one of three conditions: (I) captured and isolated (n = 11), (II) captured and paired with a male (n = 9) and (III) virgin and isolated (n = 12). After two days on-host the mites were removed from the capsules and eggs were incubated as described above. Resulting protonymphs were reared to adulthood and sexed.

### Experiment 2: characterizing the effect of mating deprivation on sex of NFM offspring

Females (n = 2) captured from an existing infestation were held for two days to allow eggs developing at the time of capture to be oviposited. Each female was then isolated in a capsule on-host (Day 0). Each female was transferred into a new capsule daily. Eggs oviposited in a capsule were collected, hatched, reared to adulthood and sexed. This process continued until the parent female died or was lost.

### Experiment 3: testing the ability of mother-son mating to produce female offspring

Individual adult males produced by virgin females (Experiment 1) were isolated with their mothers (n = 9) for 7–14 days on-host to provide opportunities to mate and reproduce. All mothers were 13 days old at the time of pairing and the sons were estimated to be 7–12 days old. The post-pairing offspring were reared to adulthood and sexed.

### Analysis

Data from Experiment 1 were analyzed using a Wilcoxon signed-rank test (Systat 12), comparing the number of each sex produced to the null expectation of an even distribution (proportion of female offspring = 0.5). Capsule Experiments 2 and 3 were proof of concept experiments and were not statistically analyzed.

## Results

### Experiment 1: determining the effect of mating opportunity on sex of NFM offspring

Captured female mites (n = 11) in isolation produced a total of 16 female and 7 male offspring (pooled SR of 2.3:1). Among the captured females, the mean (±SE) proportion of offspring that was female was 0.665 (±0.135), which did not differ from the null expectation of an even sex ratio (Wilcoxon Z = −1.812, P = 0.070) (Table 1). Captured females (n = 9) paired with males produced a total of 15 female and 3 male offspring (pooled SR of 5:1). Among females paired with males, the mean (±SE) proportion of offspring that were female was 0.806 (±0.116), which was significantly female biased (Wilcoxon Z = −2.209, P = 0.027). Virgin females (n = 12) produced a total of 66 male offspring and no female offspring, which was decidedly male biased (Wilcoxon Z = 3.276, P = 0.001).

**Table 1:** Offspring produced by female NFM isolated in capsules on-host

**Reproducing Female**		**Offspring**			**Mean (±SE)**	
**n**	**Source**	**Isolation**	**# Female**	**# Male**	**Proportion Female**	**P-value**
11	Captured	Alone	16	7	0.665 (±0.135)	0.07
9		With Male	15	3	0.806 (±0.116)	0.027
12	Raised (virgin)	Alone	0	66	0	0.001
*9		With Son	6	5	0.444 (±0.056)	-------

* Of 9 virgin females paired with a son, only 3 produced daughters that are represented by the offspring summary statistics. P-values were produced by Wilcoxon signed-rank tests of offspring sex ratio against null expectation of 1:1 ratio.

### Experiment 2: characterizing the effect of mating deprivation on sex of NFM offspring

One female produced eggs over 20 days and died after 22 days. She produced eggs that developed into daughters at 6, 13, and 14 days post isolation, and produced an egg that developed into a son at day 12. The other female produced eggs over 12 days, before she was lost on day 15. She produced eggs that developed into daughters 2, 3, 6, 7, and 11 days post isolation. She did not produce sons.

### Experiment 3: testing the ability of mother-son mating to produce female offspring

Three out of nine virgin females produced female offspring, after being paired with a son. One female produced 3 daughters and 3 sons. Another female produced 1 daughter and 2 sons. The final female produced 1 daughter and 1 son. All remaining females produced only male offspring.

## Discussion

The NFM is a cosmopolitan ectoparasite of birds and an economic pest of the poultry industry in the United States [1,2]. Populations of NFM are typically female-biased and past karyotyping suggested the NFM has haplodiploid sex determination [4,7]. This study determined that male NFM are produced from unfertilized eggs (arrhenotoky) and that virgin females are able to produce and mate with sons (oedipal mating) to subsequently produce female offspring. These observations are relevant to the epidemiology of NFM outbreaks in commercial poultry flocks. For sexually reproducing parasites to establish on a new host the dispersing females must be mature and mated, or must colonize a new host with males [8,10]. Transmission of NFMs between hosts begins early during an infestation when there are relatively few mites (low mating opportunity) and many individuals may be immature [18]. Oedipal mating provides a way for female NFMs to disseminate to new hosts without being constrained by mating opportunity, or sexual maturity. This makes all females, regardless of stage, capable of establishing on a new host. For an obligate parasite this high transmission potential represents a tremendous ecological advantage [9].

Mated females in isolation were able to produce daughters for up to 14 days [19]. Under natural conditions mated NFMs may produce daughters over a longer period. The ability of females to store sperm could also enhance the transmission potential of NFMs. Sperm storage reduces the necessity for males since females may not need repeated insemination throughout reproductive maturity. As a result, the population can persist with a strong female-biased sex ratio (i.e. a maximum number of transmissible individuals). Although NFMs reside on the host bird for the entire life cycle, they are able to survive off-host for up to 35 days [20]. The ability to store sperm extends the capacity of dispersing NFMs to establish new infestations.

The reproductive biology of NFM is also informative to basic studies of arrhenotoky, mate competition and adaptation. Cruickshank and Thomas [11] tested the hypothesis that arrhenotoky evolves from a pseudo-arrhenotokous ancestral state, using a phylogenetic study of Mesostigmatid mites. Our determination that NFM, a member of this group, is arrhenotokous supports their conclusion. Female biased sex ratios can be explained by conditions of local mate competition, where small, isolated populations founded by a few females produce selection for a female-biased sex ratio [21]. This has empirical support from studies of the spider mite, which is also haplodiploid and arrhenotokous [22,23]. Populations of NFM are produced by few founders and are aggregated on the host body [18]. Thus, NFM may represent another system where the effects of local mate competition have shaped reproductive biology. Finally, haplodiploidy provides a hypothetical advantage for adaptation because deleterious alleles may be lost rapidly through males [24,25]. The NFM has demonstrated a remarkable ability to adapt to different host species [16] and pesticide pressure [3]. Here too, the NFM may prove a useful taxon to explore the basic evolutionary implications of haplodiploidy.

In other haplodiploid mite systems (e.g. spider mites) it is possible for mated females to selectively adjust the sex ratio of offspring, depending on resource availability and mating opportunities [5,23]. It is not clear from our study if NFM females actively manipulate sex ratio. Captured females paired with males produced more daughters than captured females in isolation. While this suggests that females in proximity to males may produce more daughters, experiments 2 and 3 indicate that NFMs do not manipulate offspring sex ratios. Previously mated females (experiment 2) isolated without a mate for an extended period of time produced strongly female-biased offspring. Pairing of virgin females with a son (experiment 3) resulted in few female offspring and a roughly balanced sex ratio. Macke *et al.*[23] noted that sex ratio control of *Tetranychus* mites changed with female age and the number of mating events. The ages and frequency of mating were unknown for the NFMs we collected from intact infestations. The sex ratios of offspring in experiment 3 may have been affected by using mites that were too old or were perhaps influenced by prolonged reproduction without males. Additional experiments will be required to determine what factors impact female versus male production by mated NFMs. Another factor that could contribute to sex ratio control in the NFM is sex-distorting endosymbionts (e.g. *Wolbachia*) [26,27]. The mites used in our study came from a colony closed to immigration for several hundred generations [2] that did not test positive by PCR screening for the endosymbionts *Wolbachia*, *Spiroplasma* or *Cardinium*. Nonetheless, it is possible that endosymbiotic bacteria could play a role in the reproductive biology of field populations of this ectoparasitic mite.

## Conclusions

The northern fowl mite is a long-standing and common ectoparasitic pest of poultry in the United States. The mite is able to spread rapidly within a flock, reaching high densities (>70,000 mites/bird) that impair bird health and productivity [1,2]. Populations of NFMs are typically female biased (80%) and the mites are haplodiploid [4,7]. We determined that unmated females produce exclusively male offspring and that mated females can produce both sexes. Importantly, unmated females are able to produce and mate with sons (oedipal mating), which then allows those females to produce both sexes. In the context of parasite ecology this reproductive system is advantageous because females are able to disseminate to new hosts and start infestations without prior mating, without males, or as juveniles. This greatly expands the transmission potential for this ectoparasite and helps to explain why it is a persistent pest.

## Competing interests

The authors declare that they have no competing interests.

## Author's contributions

Both authors participated in the conceptualization of the study. JM conducted the laboratory experiments. Both authors analyzed the data, drafted the manuscript, participated in revision of the manuscript and approved the final, submitted version of the manuscript.
